# Physiologic Adaptation to Macronutrient Change Distorts Findings from Short Dietary Trials: Reanalysis of a Metabolic Ward Study

**DOI:** 10.1016/j.tjnut.2023.12.017

**Published:** 2023-12-19

**Authors:** Adrian Soto-Mota, Lisa T. Jansen, Nicholas G. Norwitz, Mark A. Pereira, Cara B. Ebbeling, David S. Ludwig

**Affiliations:** 1 Metabolic Diseases Research Unit. National Institute of Medical Sciences and Nutrition Salvador Zubiran. Mexico City, Mexico; 2 Tecnologico de Monterrey. School of Medicine. Mexico City, Mexico; 3 Department of Dietetics & Nutrition, University of Arkansas for Medical Sciences, Little Rock, AR, United States; 4 Arkansas Children’s Nutrition Center, Department of Pediatrics, University of Arkansas for Medical Sciences, Little Rock, AR, United States; 5 Harvard Medical School, Boston, MA, United States; 6 Division of Epidemiology and Community Health, School of Public Health, University of Minnesota, MN, United States; 7 New Balance Foundation Obesity Prevention Center, Boston Children’s Hospital, Boston MA, United States; 8 Department of Nutrition, Exercise and Sports, University of Copenhagen

**Keywords:** obesity, low-carbohydrate diet, low-fat diet, macronutrients, insulin, body composition, clinical trial, methodology

## Abstract

An influential 2-wk cross-over feeding trial without a washout period purported to show advantages of a low-fat diet (LFD) compared with a low-carbohydrate diet (LCD) for weight control. In contrast to several other macronutrient trials, the diet order effect was originally reported as not significant. In light of a new analysis by the original investigative group identifying an order effect, we aimed to examine, in a reanalysis of publicly available data (16 of 20 original participants; 7 female; mean BMI, 27.8 kg/m^2^), the validity of the original results and the claims that trial data oppose the carbohydrate–insulin model of obesity (CIM). We found that energy intake on the LCD was much lower when this diet was consumed first compared with second (a difference of −1164 kcal/d, *P* = 3.6 × 10^-13^); the opposite pattern was observed for the LFD (924 kcal/d, *P* = 2.0 × 10^-16^). This carry-over effect was significant (*P* interaction = 0.0004) whereas the net dietary effect was not (*P* = 0.4). Likewise, the between-arm difference (LCD - LFD) was −320 kcal/d in the first period and +1771 kcal/d in the second. Body fat decreased with consumption of the LCD first and increased with consumption of this diet second (−0.69 ± 0.33 compared with 0.57 ± 0.32 kg, *P* = 0.007). LCD-first participants had higher β-hydroxybutyrate levels while consuming the LCD and lower respiratory quotients while consuming LFD when compared with LFD-first participants on their respective diets. Change in insulin secretion as assessed by C-peptide in the first diet period predicted higher energy intake and less fat loss in the second period. These findings, which tend to support rather than oppose the CIM, suggest that differential (unequal) carry-over effects and short duration, with no washout period, preclude causal inferences regarding chronic macronutrient effects from this trial.

## Introduction

Does macronutrient composition, and especially the ratio of dietary fat to carbohydrate, affect body weight over the long term in the general population? The answer to this question has major significance to the scientific understanding of obesity pathogenesis and to public health prevention efforts. However, despite more than a century of research, the effects of macronutrient composition on body weight remain an unresolved controversy.

Most clinical studies of macronutrients and body weight employ 1 of 2 designs, both of which have strengths and limitations [1,2]. Observational studies can examine long-term relationships involving diet, or change in diet, in large groups of people consuming habitual diets, conferring potential relevance to public health prevention. However, these studies suffer from a variety of methodologic concerns, importantly including dietary assessment errors, confounding, and reverse causality. Long-term behavioral trials, in which participants can be randomly assigned to 2 or more contrasting diets for many months or years, reduce (but do not eliminate) the risk for confounding and other biases. These trials typically rely on low-intensity interventions, such as group nutrition education and dietary counseling with limited follow-up and process measures, which do not elicit strong, sustained differentiation in diet between treatment groups. Not surprisingly, they tend to show limited long-term efficacy of all types of diets for weight loss [3].

With the provision of prepared meals and rigorous control of potential confounders, residential feeding studies aim to produce high-quality evidence to support causal inference. However, due to pragmatic and cost considerations, these trials are characteristically short (i.e., ≤2 wks) and have other methodologic and design concerns that may limit long-term extrapolation of the acute effects seen in these studies among the general population.

In a recent metabolic ward feeding study [4], 20 participants were randomly assigned to an isocaloric LCD or LFD using a cross-over design, with 2-wk diet arms and no washout period. Mean energy intake and fat mass were greater for individuals on the LCD, findings interpreted as opposing the carbohydrate–insulin model (CIM) of obesity [[5], [6], [7], [8], [9]]. In the CIM, a high-glycemic load diet (with large amounts of rapidly digestible carbohydrate) increases the insulin-to-glucagon ratio in the postprandial phase, altering substrate partitioning from oxidation in lean body organs toward deposition in adipose tissue. Thus, overeating—and the positive energy balance that must accompany weight gain—results from, not causes, increasing adiposity. For this reason, the CIM targets high-glycemic load carbohydrates, rather than total calorie intake, for the prevention and treatment of obesity.

In the original article [4], the authors reported that no carry-over effect was present, and therefore its potential influence was not considered in data interpretation. The absence of a carry-over effect contrasts with several other reports in which prolonged physiologic changes, lasting several weeks or longer, were observed after major changes in macronutrient intake [[10], [11], [12]]. At a recent scientific conference [13] and in an online preprint [14], the original investigative team presented evidence to suggest that a carry-over effect did, in fact, exist. With the benefit of this amended information, we undertook a reanalysis of available individual participant data to determine whether the primary findings remain valid.

## Trial Intervention and Original Results

For the trial (NCT03878108), 21 adults were randomly assigned to “animal-based, ketogenic” or “plant-based, low-fat” diets on an inpatient research unit for 2, 2-wk diet periods in a cross-over design, without an intervening washout period. One participant withdrew, leaving 20 for the main outcome analyses (9 female; mean age, 29.9 y; mean BMI, 27.8). Macronutrient composition, as a proportion of energy, for the provided LCD and LFD were carbohydrates 10% compared with 75% and fat 76% compared with 10%, respectively, with ∼14% protein on both diets. In addition to the provided foods, participants were allowed to eat ad libitum from the assigned diets. The main outcome of the trial was total energy intake, which was greater when consuming the LCD (a difference of 689 kcal/d, *P* < 0.0001).

## Assessment of Publicly Available Trial Data

We obtained individual participant data from Open Science Framework [Hall KD. Data and SAS code [Internet]. OSF; 2022. Available at https://osf.io/zdwqb/]. Due to participant dissent to data sharing, most of the data from 4 participants included in the original research analysis were deleted from the registry, decreasing the statistical power of our reanalysis to some degree. The remaining 16 had similar baseline characteristics to the original 20, as shown in Table 1. Calculation of the energy intake outcome with these 16 participants, disregarding carry-over effects, yields similar results to the original report (a diet effect of 695 kcal/d).

**TABLE 1:** Baseline characteristics among participants in the original report and the available dataset

Participant characteristics	Original report (*n* = 20)	Available dataset (*n* = 16)
Male/female (*N*)	11/9	9/7
Age (y)	29.9 ± 6.4	29.4 ± 6.9
Height (m)	1.71 ± 0.09	1.70 ± 0.09
Body weight (kg)	80.8 ± 18.2	80.8 ± 20.2
BMI	27.8 ± 5.9	27.8 ± 6.4
Fat mass (kg)	26.9 ± 11.2	26.8 ± 12.1
Body fat (%)	32.8 ± 9.8	32.6 ± 10.0
Resting energy expenditure (kcal/day)	1550 ± 287	1582 ± 282

Data are means ± SD.

Original report data are from Hall et al. [4].

Carry-over effects were tested with a Diet∗Period interaction term in a linear model. As part of the sensitivity analyses (and to account for the longitudinal design of the original study), an adjusted linear-mixed effects model with individual slopes was used as well. We used R version 4.0.3 with R Studio version 2023.06.01-Build 524 for all analyses, including packages updated to their latest version as of September 2023. Data are expressed as mean ± SE unless otherwise indicated. No additional institutional review board approval (beyond that described in the original trial paper) was required for this reanalysis of publicly available data [4].

## Energy Intake and Body Composition

**FIGURE 1:**
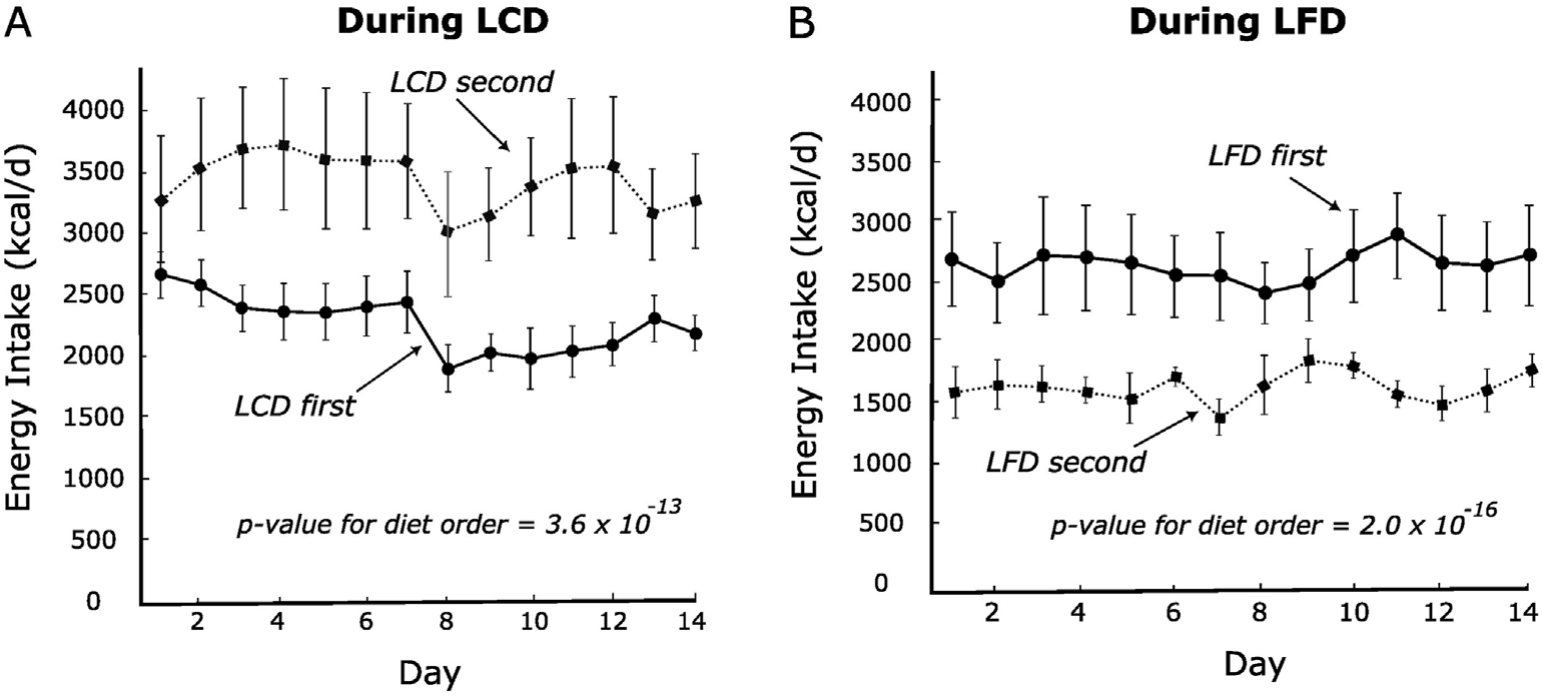
Daily energy intake on test diets by diet order. (A) Low-carbohydrate diet. (B) Low-fat diet. Solid line, the diet came first; dotted line, the diet came second *n* = 16.

**FIGURE 2:**
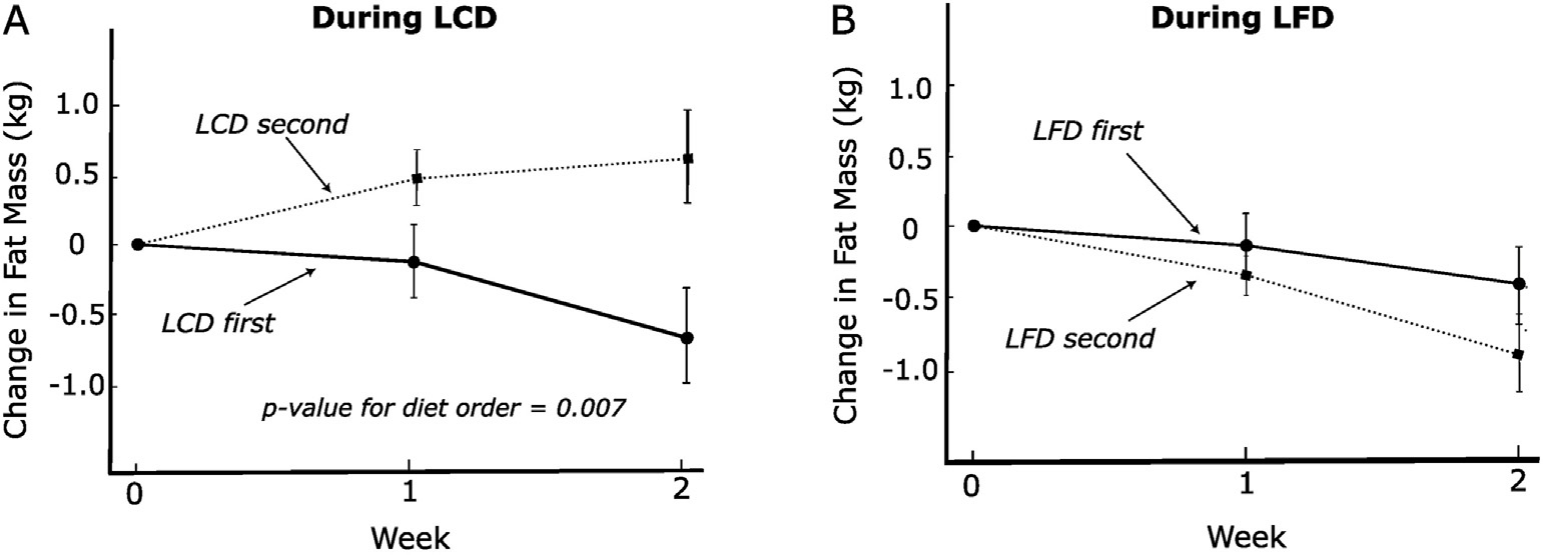
Change in fat mass on test diets by diet order. (A) Low-carbohydrate diet. (B) Low-fat diet. Solid line, the diet came first; dotted line, the diet came second *n* = 16.

In a repeated measures model using between-participant comparisons, mean energy intake on the LCD was much lower when this diet came first compared with second (a difference of −1164 kcal/d, *P* = 3.6 × 10^-13^), as depicted in Figure 1. Conversely, mean energy intake on the LFD was much higher when this diet came first (924 kcal/d, *P* = 2.0 × 10^-16^). Qualitatively consistent results were obtained with the use of a linear-mixed effects model (see publicly available code). Likewise, the between-arm difference (LCD - LFD) was −320 kcal/d in the first period and +1771 kcal/d in the second. Similarly, body fat decreased with consumption of LCD first and increased with consumption of the LCD second (−0.69 ± 0.33 compared with 0.57 kg ± 0.32, *P* = 0.007), as depicted in Figure 2.

In univariate linear models that do not consider carry-over effects, LCD order predicted both outcomes substantially better than diet composition (LCD compared with LFD). The *R*^*2*^ for LCD order was more than double that for diet composition (energy intake *R*^*2*^ = 29% and 12%; fat loss *R*^*2*^ = 8% and 3%, respectively). To address carry-over effects, we conducted a linear model interaction test on energy intake. This model showed an exceptionally large carry-over effect (2090 kcal/d for the between-period difference in the dietary effect, *P* = 0.0004), whereas the net diet effect was not significant (*P* = 0.4). These findings were not materially different in a linear-mixed-effects model with individual slopes and adjustment for potential confounders including baseline BMI, sex, and resting energy expenditure.

## Ketogenesis and Respiratory Quotient

**FIGURE 3:**
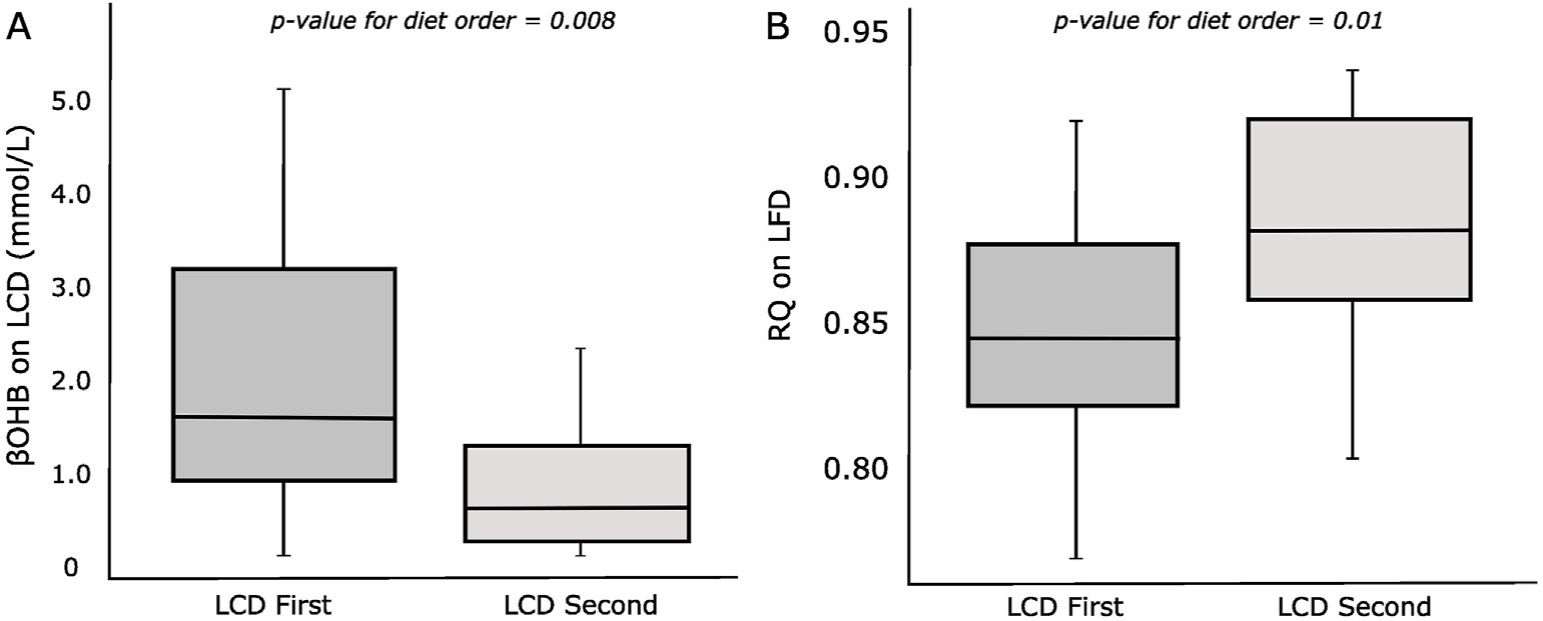
Metabolic variables on test diets by diet order. (A) Blood β-hydroxybutyrate concentration during the low-carbohydrate diet. (B) Respiratory quotient during the low-fat diet. *P* values obtained with a univariate linear model *n* = 16.

The existence of a carry-over effect, indicated by the analyses above, provides an opportunity to examine associated metabolic adaptations. β-hydroxybutyrate (βOHB) is the dominant serum ketone body and a critical component of metabolic adaptation on a carbohydrate-restricted (ketogenic) diet. Participants who consumed the LCD first compared with second achieved significantly higher βOHB blood concentrations on the LCD (*P* = 0.008). In addition, the LCD-first compared with second participants had significantly lower respiratory quotient (RQ), indicative of greater fat oxidation, on the LFD (*P* = 0.01) (Figure 3). However, these analyses did not distinguish whether the metabolic changes caused, resulted from, or otherwise interacted with differences in energy intake by diet order.

## Prediction of Energy Intake and Fat Mass on Diet 2 by Insulin Secretion on Diet 1

**FIGURE 4:**
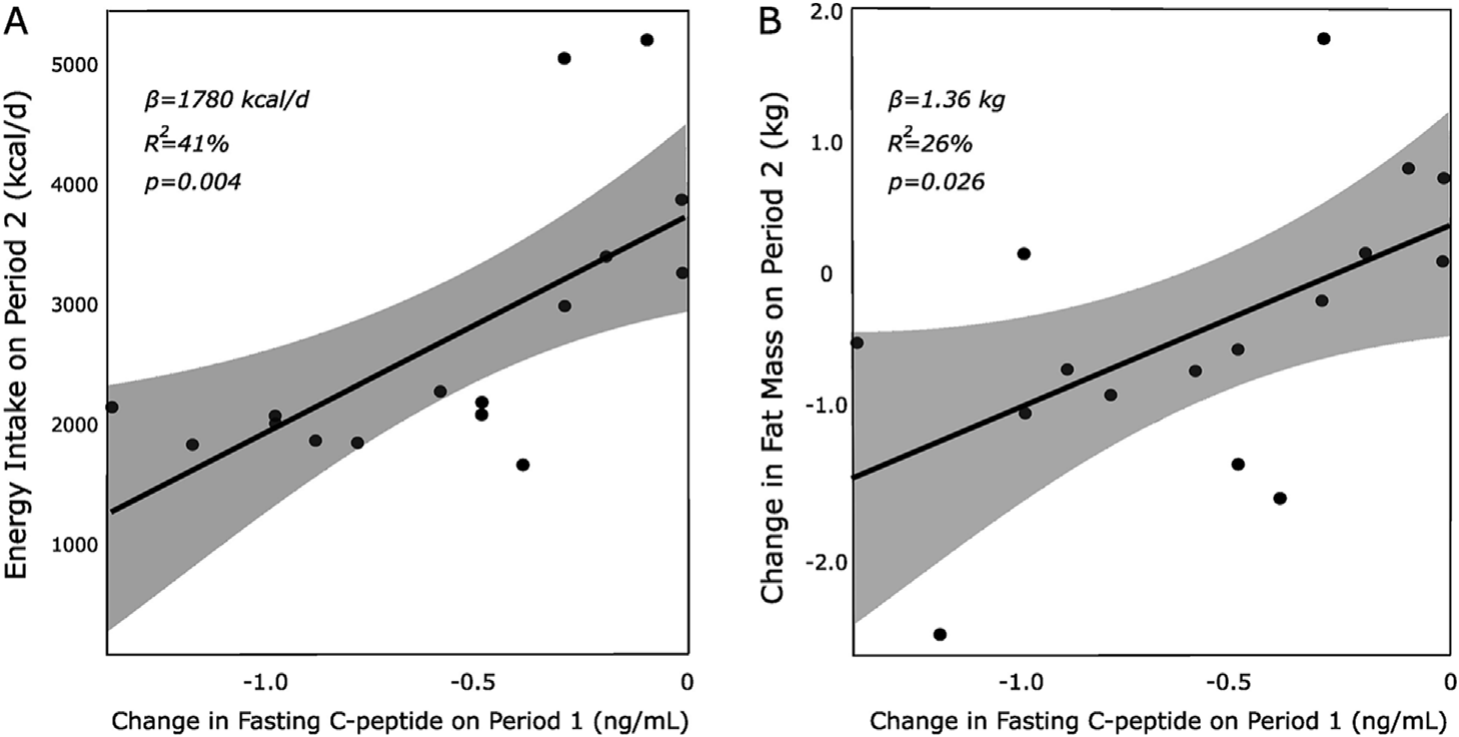
Prediction of second diet period outcomes by first diet period change in C-peptide. (A) Mean energy intake. (B) Change in fat mass. *P* values obtained with a univariate linear model. *n* = 16.

The CIM hypothesizes that high insulin secretion promotes substrate partitioning into adipose tissues, driving increased adiposity and the positive energy balance that must accompany it. Because macronutrient composition may affect insulin clearance, and therefore serum concentration [15], we used C-peptide to test this hypothesis. Changes in C-peptide concentration, a measure of insulin secretion, from baseline to the end of the first diet period were associated with ad libitum energy intake [β = 1780 kcal/d (95% CI: 750, 2811 kcal/d) per ng/mL change in C-peptide; *R*^*2*^ = 0.41; *P* = 0.004] and change in fat mass [β = 1.36 kg (95% CI: 0.3, 2.5 kg) per ng/mL change in C-peptide; *R*^*2*^ = 0.26; *P* = 0.026] during the second diet period, with larger decreases in C-peptide predicting favorable responses (Figure 4).

## Implications to Causal Inference in the Original Trial

Many dietary and environmental factors affect body weight over the short but not long term, ranging from severe calorie restriction to the size and color of dinner plates [16]. However, these transient influences, whether psycho-behavioral or physiologic in origin, contribute little to the understanding of obesity pathogenesis or the design of more effective public health intervention. Our analyses suggest that the main effects in the original trial report [4] suffered from this problem, highlighting the pitfalls of short-term feeding studies despite their ability to achieve rigorous control over several key experimental conditions.

We found that the order in which the diets were consumed had a dominant effect on study outcomes, dwarfing those of the diets themselves. To conceptualize this problem, one can consider the LCD and LFD as each comprising 2 different exposures. The LCD that came first produced 1164 kcal/d less food intake than the one that came second, opposite to the pattern observed with the LFD (924 kcal/d more for the first compared with the second period). These findings demonstrate the presence of a major *differential* (unequal) carry-over effect.

The nature of a carry-over effect has fundamental implications for causal inference. One common form of carry-over effect is nondifferential. That is, regardless of the order of the treatments, participants will consistently respond either better or worse in period 1 than in period 2. This scenario may arise with learning (better response in 2, after practice) or boredom (worse in 2). This type of carry-over can be identified using the within-individual comparisons inherent to a cross-over trial. Critically, bias in this situation can be distinguished from the treatment effect and prevented by counter-balancing random treatment assignment.

A more concerning type of carry-over effect is differential, in which the treatments have unequal effects upon each other. In the most severe case, the residual effects of one treatment may be opposite to that of another treatment, as evidently occurred in the trial [4]. Differential carry-over comprises a potentially fatal threat to the validity of cross-over studies, especially in the 2 × 2 design employed here [[17], [18], [19], [20], [21], [22], [23], [24], [25], [26], [27], [28], [29]]. The problem, according to Wang and Hung [29], is that “(differential) carry-over effects are completely confounded with treatment-by-period interaction and sequence effects.” Consequently, according to Hill and Armitage [23], “the task of disentangling treatment effects from both time and carry-over effects from the previous treatment period can be difficult or even impossible.” Highlighting this central methodologic limitation, Putt [26] observed:

“In a cross-over study, the test for the treatment effect draws efficiency from the within-subject comparison. In contrast, the test for carry-over is based on the between-subject contrast and has poor power relative to the test for the treatment effects. Should carry-over occur, this low power makes it difficult to detect.”

Because of this limitation, many repetitions of a trial may be required to identify even relatively large differential influences (e.g., 25%–50% of the treatment effect) [26]. In practice, a washout period is used to minimize the risk of clinically relevant biases. However, here, the carry-over effect can be clearly distinguished using the insensitive between-participant comparisons due to its massive magnitude (multiples of the putative treatment effect). Because this effect cannot be separated from the treatment effect, the conditions for causal inference in the trial [4] were not met and the LCD compared with LFD comparison for the main outcomes are not valid.

With a major differential carry-over effect as occurred here, the only valid test is the between-participant comparison in the first treatment period, discarding the biased data from the second period [17,18,23,28,30]. Although underpowered, these tests suggest an advantage for the LCD compared with LFD, with results opposite from the original report for energy intake averaged across both weeks (2251 compared with 2570 kcal/d, *P* = 0.36), energy intake in week 2 (2061 compared with 2582, *P* = 0.0003) and change in total fat mass (−.043 compared with −0.29 kg, *P* = 0.75), respectively.

## Physiologic Mechanisms Underlying Unequal Carry-over Effects

A substantial change in macronutrient consumption elicits myriad adaptations in hormone secretion, gene expression, tissue-specific metabolic pathways, and psycho-behavior responses [[31], [32], [33], [34], [35], [36], [37], [38]]. This adaptive process seems to require several weeks to months before reestablishment of homeostasis on the new diet according to extensive animal [36,[39], [40], [41], [42]] and human [34,37,38,[43], [44], [45], [46], [47], [48], [49], [50]] evidence. During this period, measurements of biological and behavioral outcomes may reflect transient phenomena rather than chronic macronutrient effects.

Prior consumption of an LCD lowers glucose tolerance, and by inference, insulin secretion and/or sensitivity during an oral glucose tolerance test, as has been observed for nearly a century [11]. In a recent study, progressive changes in several measures of glucose homeostasis were observed for 1 to 2 mo after changing from a ketogenic LCD diet to high-carbohydrate diets [11]. Conversely, the process of adapting to an LCD among people habituated to a high-carbohydrate diet seems to require ≥2 to 3 wk, and probably substantially longer. In a meta-analysis of LCD feeding studies, total energy expenditure decreased slightly among trials lasting <2.5 wk but increased substantially among longer trials [12]. Additional evidence of this transient adaptive process is the waning difference in daily energy intake between diets in the original trial report from the first to second wk (of both periods) which, if extrapolated linearly, would extinguish after another 2 wk [4].

## Implications to the Carbohydrate–Insulin Model

Despite the inherent trial limitation, our findings tend to support, rather than oppose, the CIM. An unbiased test of energy intake limited to week 2 of the first diet period (i.e., after 1 wk of metabolic adaptation), showed a substantial and statistically significant advantage for the LCD (521 kcal/d less energy intake, *P* = 0.0003). Furthermore, previous consumption of LCD had a prolonged beneficial effect during the subsequent LFD period, with lower energy intake and RQ. In contrast, prior consumption of the LFD had a prolonged adverse effect on the subsequent LCD period, with increased energy intake and adiposity, and reduced ketogenesis. In addition, a measure of insulin secretion on the first diet explained a remarkable amount of heterogeneity in food intake (41%) and changes in fat mass (26%) on the second diet as hypothesized in the CIM, considering the many within- and between-individual sources of variability in these outcomes and measurement imprecision.

## Conclusions

In summary, high-quality evidence from complementary lines of investigation will be needed to resolve long-standing debates regarding macronutrient composition and obesity, and test the CIM. Feeding studies may contribute significantly to this evidence, but these must be of sufficient duration to distinguish transient adaptive responses from chronic effects.

Our findings raise concern about the new NIH initiative on precision nutrition, which plans a major investment into cross-over feeding trials with 2-wk diet periods (clinicaltrials.gov identifier NCT05701657). Although these trials will incorporate a washout period, the minimum specified duration of 2 wk does not appear sufficient to exclude major bias from carry-over effects. Beyond carry-over effects, findings from 2-wk diet periods seem unlikely to elucidate the long-term metabolic effects of macronutrients, or to inform choice of diet in the clinical and public health arenas.

For all trials, outcomes such as energy intake and expenditure should be collected after ≥1 to 2 mo on test diets, based on available data on the timeframe of metabolic adaptation to major changes in macronutrients [11,12,34,38,[43], [44], [45],^50^]. For cross-over trials, a washout period of ≥3 mo should be incorporated, to allow carry-over effects to dissipate. Measurement of body composition, reflecting cumulative effects during and after adaptation, requires even longer study, ≥6 mo. Admittedly, such trials involve major logistic and other challenges for researchers and participants. To reduce these barriers, creative study methods should be developed, such as collaborations with residential educational institutions, the military, and perhaps, given appropriate ethical protections, prisons. Finally, our reanalysis, as enabled by the original investigators [4], highlights the importance to scholarly discourse of open access to full trial data.

## Acknowledgments

We thank Walter Willett for thoughtful review of the manuscript and Shui Yu for verifying the data analyses and statistical code. LTJ was supported by the Center for Childhood Obesity Prevention, funded by the National Institute of General Medicine Sciences of the National Institutes of Health under Award Number P20GM109096 (Arkansas Children’s Research Institute, PI: Weber).

## Author contributions

The authors’ responsibilities were as follows – AS-M, LTJ, NN, CBE, DSL: designed the analyses; AS-M, LTJ: conducted the data analysis; MAP: helped with statistical analyses and interpretation; AS-M, LTJ, NN, MAP, CBE, DSL: interpreted the data and participated in manuscript preparation; and all authors: read and approved the final manuscript.

## Conflicts of interest

NN is coauthor of a Mediterranean low-carbohydrate diet cookbook; he donates all royalty payments to nutrition research and education. DSL received royalties for books on obesity and nutrition that recommend a low-glycemic load diet and his spouse has ownership of a nutrition consulting business. All other authors report no conflicts of interest.

## Funding

The authors reported no funding received for this study.

## Data availability

The analysis code is available at: https://github.com/AdrianSotoM/DietOrder.
